# The rollout of Community ART Refill Groups in Zimbabwe: a qualitative evaluation

**DOI:** 10.1002/jia2.25393

**Published:** 2019-08-27

**Authors:** Aaron F Bochner, Elizabeth Meacham, Nathan Mhungu, Phibion Manyanga, Frances Petracca, Claudios Muserere, Gloria Gonese, Batsirai Makunike, Blessing Wazara, Clorata Gwanzura, Ponesai Nyika, Ruth Levine, Tsitsi Mutasa‐Apollo, Shirish Balachandra, Stefan Z Wiktor

**Affiliations:** ^1^ International Training and Education Center for Health (I‐TECH) Department of Global Health University of Washington Seattle WA USA; ^2^ International Training and Education Center for Health (I‐TECH) Harare Zimbabwe; ^3^ Ministry of Health and Child Care Harare Zimbabwe; ^4^ U.S. Centers for Disease Control and Prevention Harare Zimbabwe

**Keywords:** HIV, ART delivery, differentiated care, differentiated service delivery, community‐based, Zimbabwe

## Abstract

**Introduction:**

Community ART Refill Groups (CARGs) are an antiretroviral therapy (ART) delivery model where clients voluntarily form into groups, and a group member visits the clinic to collect ART for all group members. In late 2016, Zimbabwe began a nationwide rollout of the CARG model. We conducted a qualitative evaluation to assess the perceived effects of this new national service delivery model.

**Methods:**

In March‐June 2018, we visited ten clinics implementing the CARG model across five provinces of Zimbabwe and conducted a focus group discussion with healthcare workers and in‐depth interviews with three ART clients per clinic. Clinics had implemented the CARG model for approximately one year. All discussions were audio recorded, transcribed, and translated into English, and thematic coding was performed by two independent analysts.

**Results:**

In focus groups, healthcare workers described that CARGs made ART distribution faster and facilitated client tracking in the community. They explained that their reduced workload allowed them to provide better care to those clients who did visit the clinic, and they felt that the CARG model should be sustained in the future. CARG members reported that by decreasing the frequency of clinic visits, CARGs saved them time and money, reducing previous barriers to collecting ART and improving adherence. CARG members also valued the emotional and informational support that they received from other members of their CARG, further improving adherence. Multiple healthcare workers did express concern that CARG members with diseases that begin with minor symptoms, such as tuberculosis, may not seek treatment at the clinic until the disease has progressed.

**Conclusions:**

We found that healthcare workers and clients overwhelmingly perceive CARGs as beneficial. This evaluation demonstrates that the CARG model can be successfully implemented on a national scale. These early results suggest that CARGs may be able to simultaneously improve clinical outcomes and reduce the workload of healthcare workers distributing ART.

## 1 Introduction

As countries work to reach the Joint United Nations Programme on HIV/AIDS Fast‐Track 90‐90‐90 targets by 2020 1, differentiated service delivery (DSD) is a widely embraced strategy 2, 3, 4, 5. As outlined in the 2016 World Health Organization guidelines on antiretroviral therapy (ART) and described by others, DSD is based upon the idea that HIV service delivery should be offered in different formats to fit the varying needs of people living with HIV (PLHIV) 6, 7. With an estimated 36.9 million PLHIV globally, 21.7 million of whom are accessing ART, significant resources are required for ART service delivery and many clients struggle to obtain their ART and achieve viral suppression 8. Differentiated ART delivery models have the potential to simultaneously improve client outcomes while reducing the workload for healthcare workers (HCWs).

The Community ART Group (CAG) DSD model was originally developed by Médecins Sans Frontières (MSF) in Mozambique 9. In the CAG model, ART clients form into groups, and group members take turns collecting ART for all members, reducing the frequency of clinic visits. Regional and national pilots in Mozambique found higher rates of retention among CAG members 10, 11, and qualitative analyses and satisfaction surveys indicated positive acceptance by ART clients and healthcare workers 12, 13, 14, 15. These findings have led several Ministries of Health to recently incorporate the CAG model into their national guidelines 2, 3, 4. However, evaluations of pilot programmes reported concerns about sustainability and noted that CAG implementation was highly dependent on MSF staff who were working at pilot facilities 12, 15. Thus, significant uncertainty remains around whether CAGs and other ART DSD models can be implemented at scale 16.

Zimbabwe has an estimated adult HIV prevalence of 13.3%, with 1.3 million PLHIV 17. In late 2016, the Zimbabwe Ministry of Health and Child Care (MoHCC) began a nationwide rollout of Community ART Refill Groups (CARGs), based on the Mozambique CAG model 2. At the time of this evaluation, programmatic data from 19 districts of Zimbabwe indicated that there were 35,810 active CARG members, representing 9% of ART clients in these districts. Using a combination of surveys, in‐depth interviews (IDIs), and focus group discussions (FGDs) with HCWs and ART clients, our objective was to evaluate the perceived effects of the CARG model for both HCWs and ART clients.

## 2 Methods

### 2.1 Study setting

Since October 2013, the International Training and Education Center for Health (I‐TECH) has supported HIV testing, care and treatment services in Zimbabwe as a recipient of the President's Emergency Plan for AIDS Relief (PEPFAR) funds in collaboration with the U.S. Centers for Disease Control and Prevention (CDC). I‐TECH works in 19 districts across five provinces of Zimbabwe, coordinating with the MoHCC to support service delivery at government facilities through a combination of training, on‐site mentorship and deployment of supplemental clinical staff.

Zimbabwe's national guidelines for HIV care and treatment have included DSD models for stable ART clients since 2015. In late 2016, as these guidelines were being revised 18, the MoHCC initiated a drive to implement the five ART refill options included in the guidelines: fast‐track (patients collect ART from the pharmacy without a clinical exam), club refill (facility‐based HCW‐led group refills), outreach (individual ART delivery through mobile outreach), family member refill (one individual collects ART for all family members) and CARGs. As an implementing partner, I‐TECH supported the rollout of these national guidelines through formal trainings and on‐site mentorship. Due to prioritization by funders, I‐TECH was asked to support the rollout of the CARG model at all facilities, while implementation of other DSD models was given lower priority. Thus, at most facilities, ART clients had the option of obtaining ART through the standard of care for stable clients, which was 3‐month individual refills, or through CARGs, while a minority of sites were implementing other DSD models.

### 2.2 The CARG model

The CARG model was designed based on the Mozambique CAG model 9, 10, although with some important differences. In both models, stable ART clients living in the same community are encouraged to self‐form into groups. A member of the group then visits the clinic and collects ART for all group members. In the Mozambique model, CAGs contained two to six clients and a CAG member visited the clinic monthly to collect ART 10. CARGs in Zimbabwe are larger, with four to twelve clients per group, and CARGs collect three‐month supplies of ART, reducing the frequency of clinic visits. While Mozambican CAG members were monitored by CD4 count and visited the clinic every six months for a clinical consultation, CARG members with suppressed viral loads (<1000 copies/mL) are only required to visit the clinic annually for a clinical consultation and viral load assessment. All members of a CARG usually visit the clinic together on the same day to receive these annual services. Before each ART refill visit, CARG members screen each other for tuberculosis and other opportunistic infections, and HCWs encourage clients to visit the clinic if they feel unwell. To join a CARG, clients must meet several eligibility criteria: at least six months on ART, a viral load <1000 copies/mL (CD4 >200 cells/mm^3^ when viral loads are unavailable), and no active opportunistic infections. Pregnant or breastfeeding women are also excluded.

### 2.3 Study design

The evaluation was implemented in March to June 2018, approximately one year after most facilities began implementing CARGs. In each of the five provinces I‐TECH supports, we randomly selected one district to include in the evaluation. Within each district, we used purposive sampling to select two facilities. The sample size was determined *a priori*; due to budget limitations and a desire to quickly feed evaluation results back to programme staff, the evaluation was limited to 10 facilities. All facilities with at least 10 active CARGs were eligible (a median 76% of facilities were eligible across the five districts). Facilities were selected to provide variation by facility type, facility size, population density and the proportion of ART clients in CARGs. The ten selected sites included two rural hospitals, six rural clinics and two urban clinics. At selected facilities, the number of ART clients ranged from 377 to 6,543 and the proportion of ART clients in CARGs ranged from 12% to 53%.

At each facility, IDIs were held with two CARG members and either one former CARG member, or if a former CARG member was not available, an individual who was eligible but declined to join a CARG. All IDIs and FGDs were conducted in English, Shona or Ndebele by an external consultant who had not previously worked on the CARG programme. CARG members with clinic visits scheduled for the week of the interviews were asked to visit the clinic on the day of the IDIs and were offered the opportunity to participate. Former and non‐CARG ART clients were recruited among those attending the facility on the day of data collection, and the external consultant approached ART clients and interviewed the first client he identified who was eligible and willing to participate; clinic staff did not select which ART clients were interviewed. Additionally, at each facility we conducted a FGD with HCWs who work with CARGs. Interview guides for the FGDs and IDIs were developed based on the evaluation objectives. After data collection at the first site, transcripts were reviewed and data collection tools were revised.

Additionally, prior to each IDI and FGD, participants completed a brief individual self‐administered survey that collected basic demographic information. The survey for HCWs participating in FGDs also elicited individual opinions on CARGs to address potential social desirability bias that might arise during focus groups. Participating ART clients were provided a transportation allowance ($1 U.S. dollar) and all participants were given a snack and beverage during the interview session.

### 2.4 Data analysis

All interviews and FGDs were audio recorded, transcribed, and translated to English. Data were coded for qualitative themes using a general inductive approach, with primary coding on expected themes and secondary coding on unanticipated discussion topics 19. All transcripts were coded by at least two analysts using ATLAS.ti (version 8); one analyst was the external consultant who conducted the interviews while a second analyst was I‐TECH staff. Survey results were double‐entered using Google Forms, and descriptive analyses were conducted using Microsoft Excel.

### 2.5 Ethical considerations

The evaluation protocol was approved by the Medical Research Council of Zimbabwe and was reviewed according to the U.S. CDC human research protection procedures; CDC was determined to not be engaged. Written informed consent was obtained from study participants, who were all aged 18 years and above.

## 3 Results

Across 10 facilities, 30 ART clients participated in IDIs: 19 CARG members, 6 former CARG members and 5 clients who declined to join a CARG (Table 1). At these same facilities, 46 HCWs participated in FGDs. They included 30 nurses, nine primary counsellors (a lay cadre who provides HIV testing and counselling services), six community linkage facilitators, and one environmental health technician (community linkage facilitators are a lay cadre while environmental health technicians are credentialed; both cadres track ART defaulters). Transcripts were coded at least twice as new themes emerged, resulting in the following final broad themes: CARG impact on HCWs, initial reactions to CARGs, patient barriers to joining CARGs, CARG membership benefits, CARG membership challenges, recruitment/registration and CARG structure/process. For each broad theme, multiple sub‐themes were identified and coded. This manuscript focuses on the themes of CARG impact on HCWs, CARG membership benefits and CARG membership challenges.

**Table 1:** Information on study participants

Participant group	Number of participants
Clients on ART (IDIs)	30
In CARGs, CARG leader	5
In CARGs, never CARG leader	14
Former CARG members	6
Declined to join a CARG	5
Healthcare workers (10 FGDs)	46
Nurses	30
Primary counsellors	9
Community linkage facilitators	6
Environmental health technician	1
Total	76

FGDs, focus group discussions; IDIs, in‐depth interviews.

### 3.1 Benefits of CARG membership for ART clients

During IDIs, the most frequently mentioned benefit of CARG membership was that joining a CARG saved ART clients time and reduced costs for clients who pay for transportation to the clinic. Fewer clinic visits allowed more time for work, either formal employment or addressing responsibilities around the home such as caregiving, housework or farm work. Time‐saving was also frequently identified as a primary motivator for joining a CARG.

*What inspired me to be a CARG member was the burden of traveling to the clinic and the money that I would use would be less*. (CARG member from IDI, male, aged 35‐44, rural clinic)

Since having sufficient time and money were barriers to obtaining ART, both clients and HCWs recognized that by facilitating ART delivery, CARGs helped clients improve their ART adherence.

*There may be other people who may be failing to raise money for transport to the clinic and they may not be able to walk the very long distance. These people might end up failing to come in for a refill. So if we are working as a team, only one person comes in and all people continue getting their [ART] supply*. (CARG member from IDI, male, aged 45‐54, rural clinic)

Psychosocial support provided during CARG group meetings was identified as an additional way CARGs improved ART adherence. CARG members reminded each other to take their medication, monitored each other's health and ART adherence, developed valued personal relationships and were motivated by other group members to stay adherent.

*We actually remind each other when to take our drugs… if you are part of a WhatsApp group and you see a message reminding people to take their drugs you are prompted to take them on time*. (CARG member from IDI, female, aged 35‐44, urban clinic)

Many CARG members also mentioned that CARG meetings served as an opportunity to share knowledge and information. Before joining a CARG, several clients mentioned that they felt isolated and had challenges obtaining information from HCWs. Their desire for additional information motivated them to join a CARG.

*Sometimes I might have a challenge, and if I am in a CARG I have people whom I can share with and get a solution quickly. If it's about taking my drugs, I might be reminded by someone or be corrected where I am doing it incorrectly*. (CARG member from IDI, female, aged 35‐44, rural clinic)

Several other benefits of CARG membership were mentioned. CARG members noted that wait times had reduced as fewer people were coming to the clinic. ART clients noted that the atmosphere at the clinic had improved because CARGs had shortened queues, noise had reduced and without long queues, clients had stopped quarrelling. CARGs also provided some clients with economic benefits. A few CARG groups had initiated income‐generating projects together, pooling resources and making investments as a group.

### 3.2 Challenges of CARG membership for ART clients

While the vast majority of ART clients and HCWs expressed positive views of CARGS, some individuals described challenges with CARG membership. Some clients chose to leave CARGs because another family member on ART, such as a child or pregnant spouse, was ineligible to join. Other clients had to leave CARGs when they themselves became pregnant. A HCW described a CARG that experienced challenges with a member not respecting other member's confidentiality. One CARG member desired more frequent discussions with HCWs, and several CARG members described challenges obtaining sufficient quantities of free condoms, which they had previously obtained at clinic visits. Migration, which is common in Zimbabwe due to poor economic conditions, can disrupt CARG operations as members leave.

*Most [clients] have no source of income… some have been reported to have gone to towns or outside the country looking for jobs. So l think it's a challenge*. (Nurse from FGD, female, aged 45‐54, rural hospital)

### 3.3 Impact of CARGs on the workload of healthcare workers

In FGDs there was a broad consensus among HCWs that CARGs had reduced their workload and improved the work environment at the clinic. A few HCWs described a temporary increase in workload when CARGs were first implemented, as both HCWs and CARG members had challenges correctly completing forms and aligning all CARG members’ ART pick‐up dates required additional work. After these initial challenges, HCWs noted that serving a group was easier and faster than serving individual clients. In addition to ART dispensing, individual visits often include discussions of other ailments and requests for other treatments, which take time though they may not be serious. Many clinics scheduled CARG visits on specific days and pulled out patient care booklets in advance. HCWs described how the reduced workload freed up time, allowing them to complete other tasks at the clinic.

*It's now easy for us since we no longer have a lot of people coming to the facility at the same time like we used to have in the past… in the afternoon we can actually concentrate on other tasks that we failed to do in the past because of a large number of clients coming in*. (Nurse from FGD, female, aged 35‐44, rural clinic)

HCWs also noted that the number of clients who defaulted on ART had declined since the inception of the CARG model, which meant that fewer clients needed to be tracked. In addition, several HCWs reported using CARGs to communicate with CARG members when necessary, for example, informing clients due for a viral load test.

*It is now quite easy for us to communicate with clients in CARGs. Let's say we want to communicate with clients that they have to come in for viral load testing or CD4 counts… we give the message to the client coming in for a refill who then passes the message to the group members. This has really assisted us*. (Primary counsellor from FGD, male, aged 35‐44, rural clinic)

Most HCWs reported little or no change to the amount of required paperwork, with a few HCWs reporting increased paperwork. Most paperwork remained unchanged because all individual patient ART files still had to be updated. Additional CARG forms and registers require time to complete, but some HCWs reported that time were saved because less client screening had to be documented. One HCW reported that the quality of documentation had improved due to CARGs because with workload reductions, staff had more time available to complete paperwork.

*Paperwork is still the same because even if one person comes with ten books you still have to document information for ten books, you are not documenting one book which represents ten people*. (Nurse from FGD, female, aged 24‐34, rural clinic)

In self‐administered individual surveys completed prior to each FGD, staff overwhelmingly (97%) reported that implementing CARGs had reduced their workload, consistent with what was reported in FGDs (Table 2).

**Table 2:** Healthcare worker perceptions of the impact of CARGs

Survey questions	N (%)^a^
How have CARGs impacted your workload?	
Significantly reduced	34 (76%)
Slightly reduced	10 (22%)
No impact	0 (0%)
Slightly increased	1 (2%)
Significantly increased	0 (0%)
How have CARGs impacted the care of clients on ART?	
Large improvement	40 (89%)
Small improvement	5 (11%)
No change/small decline/large decline	0 (0%)
Should continue to be supported at your facility?	
Strongly agree	35 (76%)
Agree	11 (24%)
Neutral/disagree/strongly disagree	0 (0%)

CARGs, Community ART Refill Groups.

a There were missing responses for the impact of CARGs on workload (n=1) and the impact of CARGs on client care (n=1).

### 3.4 Impact of CARGs on quality of care

The consensus among CARG members was that being in a CARG had improved the quality of care they received from the clinic. Although clients were visiting the clinic less often, no one described this as a problem since they felt empowered to visit the clinic whenever they needed. Several CARG members stated that they saw no reason to visit the clinic as long as they were feeling well.

*The number of visits has gone down, if I am not sick or would [not] want any [laboratory] testing done, there is no need to come to the clinic*. (CARG member from IDI, female, aged 35‐44, rural clinic)

When a CARG member was sick, they supported and encouraged that individual to come into the clinic. Multiple CARG members commented that they felt the quality of care they received at the clinic had improved because clinicians were less busy, allowing clinicians to provide more attention to those clients visiting the clinic.

*The nurses are actually serving us wholeheartedly because they are not under pressure*. (CARG member from IDI, female, aged 35‐44, rural clinic)

HCWs reported feeling that overall quality of care for CARG members had improved. They saw the communal support provided through CARG membership as a major benefit for clients. Multiple HCWs noted that not having large numbers of ART clients gathering together at the clinic should reduce the transmission of communicable diseases such as tuberculosis. HCWs reported being able to provide better care to those clients who came into the clinic because they were less busy.

*I think the quality of patient care has actually improved, let's take for example if one comes in with some ailment you have to examine that patient… So if they come in as an individual you take ample time to examine the client without any pressure*. (Nurse from FGD, female, aged 25‐34, rural clinic)

Although no HCWs felt CARGs were detrimental to ART clients overall, some HCWs identified specific ways CARGs could put clients at risk. Some HCWs were concerned about the level of responsibility and power that resided with CARG leaders given their limited training. Because CARG members were sharing information with each other, there was a concern that inaccurate information and rumours could spread among CARG members. Some HCWs also worried that ailments such as tuberculosis that begin with minor symptoms may be ignored by clients until the disease has progressed.

*[For] cough lasting any duration we should do a TB investigation. Those clients are not coming in, they come when they are seriously ill. They usually keep their mild conditions at their homes*. (Nurse from FGD, female, aged 45‐54, rural hospital).

In individual surveys, HCWs unanimously felt that CARGs improved the care of clients on ART, with 89% reporting a large improvement (Table 2).

### 3.5 Sustainability of the CARG model

In individual surveys, all HCWs believed CARGs should continue to be implemented in the future (Table 2). Additionally, no current CARG members expressed a desire to leave their CARG. When asked what will happen with CARGs in the future, most HCWs felt that enrolment in the CARG model would expand and that CARGs would persist because they benefit both HCWs and clients. However, several HCWs commented that CARGs require ongoing HCW support as well as sufficient supplies of ART to be sustained.

*The future of CARGs is so bright as long as we continue to get enough supplies of antiretroviral drugs*. (Nurse from FGD, female, aged 25‐34, rural clinic)

## 4 Discussion

We found that both HCWs and ART clients overwhelmingly perceived CARGs as beneficial. CARGs reduced the burden of ART delivery for patients and HCWs, while supporting ART adherence and improving the quality of life for CARG members. With tens of thousands of Zimbabwean ART clients in CARGs, and many more joining CAGs in other sub‐Saharan African countries 2, 3, 4, 5, it is critical to evaluate the early effects of CARGs on clients and HCWs.

This evaluation suggests that the CARG model can be successfully implemented on a broad scale with lower levels of external support than previous pilot studies. Unlike past pilot programmes, facilities included in this evaluation had no staff dedicated to implementing CARGs and lacked the resources to routinely conduct community visits to CARG members 13, 15, 20, 21. Yet, our results are consistent with findings from evaluations of pilot programmes 12, 20, 22, and these results bode well for other countries implementing the CAG/CARG model nationally.

Both clients and HCWs consistently self‐reported that CARGs improved adherence and reduced client loss to follow‐up. This is consistent with quantitative evaluations of pilot programmes, which found that clients in CAGs had lower rates of loss to follow‐up 11, 22, 23, 24. Once sufficient person‐time in CARGs has accrued, quantitative analyses should be done to evaluate the impact of CARG membership on retention and viral suppression. This evaluation was designed and implemented before many sites had implemented CARGs for one year, and was intended to evaluate the perceived effects of the early stages of CARG implementation. Qualitative and quantitative analyses have different limitations, so triangulating results from both approaches will provide the best evidence of the impact of CARGs.

Although perceptions of CARGs were overwhelmingly positive, participants raised some concerns that warrant further investigation. CARG leaders hold significant responsibilities, and the frequency of training they require should be evaluated to avoid misinformation that could adversely impact patients. Additionally, concern that CARGs might lead to the late presentation of diseases such as tuberculosis should be investigated. It is important to ensure that the CARG model provides access to all services and items available at the clinic, such as condoms and other family planning methods. Migration was also mentioned several times as a challenge. The fact that CARGs have more members than the original CAG model may have helped to mitigate the impact of members migrating and exiting CARGs. Lastly, with only 9% of ART clients in CARGs at the time of this evaluation, the model may not be appropriate for all types of facilities and other DSD models may have greater appeal to clients in some settings.

One limitation of this evaluation is that although the CARG model was implemented nationally, sites included in this evaluation were among the 345 government clinics receiving I‐TECH support. I‐TECH district staff provided training to HCWs on CARG implementation, and they conducted monthly mentorship visits to clinics, although these visits primarily focused on matters other than CARGs. Additionally, I‐TECH deployed HCWs to some of these facilities who were integrated into the management structure at the facility. Staff deployment did not coincide with the rollout of CARGs, and no staff were dedicated to CARG implementation. Yet it is possible that the additional human resource support was an important factor for successful CARG implementation. To scale the CARG model without implementing partner support, a Ministry of Health would need to assume I‐TECH's role providing mentorship and training around the start‐up of CARG implementation and ensure that facilities have CARG forms and sufficient HCWs to handle the temporary increase in workload associated with starting CARG implementation. The Zimbabwe MoHCC does not yet collect national data on CARG implementation, so we do not know how many CARGs have been formed in areas not receiving I‐TECH support. Additionally, it is important to note that in Zimbabwe only clinically stable clients are eligible to join CARGs, so all data presented in this manuscript are specific to stable ART clients.

## 5 Conclusions

The mix of surveys, IDIs and FGDs with ART clients and HCWs demonstrated that CARGs were regarded as highly beneficial. Future evaluations are needed to ensure that the positive views of CARGs are sustained over time, and quantitative analyses will be needed to confirm that CARGs are improving patient outcomes. Overall, these early results from implementation appear promising and suggest that CARGs may promote the objectives of DSD models by simultaneously improving patient care and reducing HCW workloads.

## Competing interests

The findings and conclusions in this article are those of the authors and do not necessarily represent the official position of the funding agencies or the Zimbabwe Ministry of Health and Child Care. The authors declare that they have no competing interests.

## Authors’ contributions

AFB, EM and FP designed the evaluation. NM conducted the interviews and focus group discussions. AB, EM, NM and FP coded the interview transcripts. PM, CM, GG, BM and BW facilitated programme management and data collection. CG and TMA supported programme implementation for the MoHCC, and PN, RL, SB and SZW provided technical oversight and support. AB led manuscript development. All authors made meaningful contributions to the manuscript content and approved the final manuscript.

## Supporting information


**Form S1.** Patient Participant Demographic Information Survey
**Form S2.** Patient Interview Guide: CARG Member
**Form S3.** Patient Interview Guide: Former CARG Member
**Form S4.** Patient Interview Guide: Non‐CARG Member
**Form S5.** Brief Satisfaction Survey for Focus Group Participants
**Form S6.** Healthcare Worker Focus Group Discussions
