# Supplementary material for: The rollout of Community ART Refill Groups in Zimbabwe: a qualitative evaluation
Source: J Int AIDS Soc. 2019 Aug 27;22(8):e25393. doi: 10.1002/jia2.25393 (PMC6711352; doi:10.1002/jia2.25393)
Supplement: Supplementary file 1 — Form S1. Patient Participant Demographic Information Survey Form S2. Patient Interview Guide: CARG Member Form S3. Patient Interview Guide: Former CARG Member Form S4. Patient Interview Guide: Non‐CARG Member Form S5. Brief Satisfaction Survey for Focus Group Participants Form S6. Healthcare Worker Focus Group Discussions [file JIA2-22-e25393-s001.pdf]

Form 1: Patient Participant Demographic Information Survey

Today's Date: ________________________ **Facility Name: ____________________________**

What is your age?

☐ 18-24 ☐ 25-34 ☐ 35-44 ☐ 45-54 ☐ 55 and above

What is your sex?

☐ Male ☐ Female

Marital Status?

☐ Single ☐ Married ☐ Divorced ☐ Widowed

CARG Status?

☐ Current CARG member ☐ Former CARG member ☐ Never in a CARG

When did you start taking ART? (Month/Year) ______________

Have you ever joined a CARG? ☐ Yes, ___________ (Month/Year) ☐ No

Are you, or have ever you been, a CARG leader/focal person? ☐ Yes ☐ No

How many people are currently in your CARG? ____________

If you are no longer in your CARG, when did you leave? (Month/Year)________________

How do you travel to the clinic?

☐ Walk ☐ Bike ☐ Kombis/Minibus/Bus ☐ Drive

How many minutes does it take you to get to the clinic from your home? _________ minutes

What does it cost you to get from home to the clinic, and back home (roundtrip costs)? _______ USD

About how long does it take you to visit a CARG member and collect your ART? __________ min

What does it cost you to visit a CARG member and collect your ART (roundtrip costs)? ______ USD

Have you ever had a viral load test done and received your results? ☐ Yes ☐ No

If yes, what was your most recent viral load? ☐ Undetectable ☐ Detectable, but <1000 ☐ ≥1000

Form 2: Patient Interview Guide: CARG Member

Instructions:

- The interview should be held in a private, quiet location.
- The interview should be conducted by a team of two: one who will lead the interview, and one who will take notes during the interview.
- A device needs to be available to record the interview, with extra batteries available in case they are needed.
- Before starting the interview, the client needs to have signed the Informed Consent Form:
  Verification of informed consent: ☐ YES ☐ NO

Introduction: Hello, my name is ____. Thank you for agreeing to talk to me today. I am here to talk with you as part of the I-TECH organization that supports the implementation of the Community ART Refill Group (CARGs) program in this district. As someone who is currently a member of a CARG, your experience and thoughts are critical to the implementation of the CARGs program. We would like to thank you for agreeing to participate in this interview. Today, I would like to talk with you briefly about your experiences being a member of a CARG, to better understand what motivated you to join a CARG, what you like/dislike about membership, the impact on your life and clinical care, as well as your suggestions for improving the CARG program.

1. To begin, I'm going to ask you a few questions about yourself and your experiences receiving care at this clinic. [*Administer Patient Participant Demographic Information Survey*]c

2. Please tell me about how you joined your CARG?
   <u>Probes:</u>
   How did you hear about CARGs?
   Why did you decide to join a CARG?
   Did you have any concerns about joining a CARG? If so, what were they?
   How was your CARG formed?

3. What is your relationship with other CARG members?
   <u>Probes:</u>
   Did you know them before joining the CARG? Friends? Family?
   How did you disclose your HIV status to other CARG members?
   How did you decide who would be the CARG leader?
   What does the CARG leader do?
   Has the CARG leader done their job adequately? If CARG leader: As a leader, what do you feel you are doing well? Are there any ways you could improve?

4. Can you describe what happens when it is time for a CARG member to visit the clinic and collect ART?
   <u>Probes:</u>
   Do CARG members meet before the clinic visit?
   If yes, how is it organized? When and where do you meet? Who keeps the client cards?
   How do you decide which CARG member conducts the clinic visit?
   Use of WhatApp?

5. Can you describe what happens after a CARG member visits the clinic to collect ART?
   <u>Probes:</u>

How are the antiretroviral therapy drugs distributed?
How has the process for distributing ART changed over time?

6. How do people deal with money within the CARG?
   Probes:
   Payment for ART (if applicable)?
   Payment for CARG leader transport?
   Other money given to CARG leader?
   Have there been any problems? How where they handled?

7. How do you think that being in a CARG has changed your ability to regularly take ART?
   Probes:
   How has being in a CARG made it easier or harder to get drugs from the clinic?
   How has being in a CARG made it easier or harder to regularly take your ART drugs?
   Have there been times other CARG members have delayed you receiving your ART?
   As a result, have you ever gone without drugs?

8. Since joining a CARG, have you had less frequent visits to the clinic to see a doctor or nurse? How do you feel about this?
   Probes:
   Is the support that you receive adequate?
   Do you have other sorts of interactions for adherence support between your visits to a doctor or nurse?
   How often do you think you would like to have some interaction with someone trained in adherence support?

9. What do you like about being in a CARG?

10. What could be changed to make CARGs better?

11. Have any individuals chosen to leave your CARG? If yes, do you know the reason(s) why?
    Probes:
    Has anyone been sick? How was it handled?

12. How did you collect ART prior to joining a CARG? How often did you pick up your ARVs?

13. Are you aware of any other options offered by your facility to reduce the number of clinic visits needed to collect your ART?
    Probes:
    After asking the open-ended question, ask the following questions. However, only ask about the differentiated ART options offered at the site you are visiting:
    - Do you know if your facility offers multimonth ART dispensing: 3 month prescriptions for ART?
    - Do you know if your facility offers Fast-track refills: facility-based individual refill from a pharmacy?
    - Do you know if your facility offers Club refills: healthcare worker-led refill groups which meet in a room at the facility every 3 months?
    - Do you know if your facility offers outreach ART services: Community-based individual ART delivered through mobile outreach every 3 months?
    - Do you know if your facility offers family member refill groups: Similar to a CARG, but all members are part of a single family?

14. Ideally, how would you like to get your ART medicine?
    Probes:
    If you could create a system for picking up your ART drugs, what would it be? How would it work?
    Frequency of visits to get ART? Frequency seen by a nurse?

Form 3: Patient Interview Guide: Former CARG Member

Instructions:

- The interview should be held in a private, quiet location.
- The interview should be conducted by a team of two: one who will lead the interview, and one who will take notes during the interview.
- A device needs to be available to record the interview, with extra batteries available in case they are needed.
- Before starting the interview, the client needs to have signed the Informed Consent Form:

  Verification of informed consent: ☐ YES ☐ NO

Introduction: Hello, my name is ____. Thank you for agreeing to talk to me today. I am here to talk with you as part of the I-TECH organization that supports the implementation of the Community ART Refill Group (CARGs) program in this district. As someone who has previously been a member of a CARG, your experience and thoughts are critical to the implementation of the CARGs program. We would like to thank you for agreeing to participate in this interview. Today, I would like to talk with you briefly about your experiences formerly being a CARG member, your opinions about membership, the impact on your life and clinical care, as well as your suggestions for improving the CARG program.

1. To begin, I'm going to ask you a few questions about yourself and your experiences receiving care at this clinic. [*Administer Patient Participant Demographic Information Survey*]

2. Please tell me about how you got started in your CARG?
   Probes:
   How did you hear about CARGs?
   Why did you decide to join a CARG?
   Did you have any concerns about joining a CARG? If so, what were they?
   How was your CARG formed?

3. What was your relationship with other CARG members?
   Probes:
   Did you know them before joining the CARG? Friends? Family?
   How did you disclose your HIV status to other CARG members?
   How did you decide who would be the CARG leader?
   What does the CARG leader do?
   Has the CARG leader done their job adequately?

4. We want to learn about how your CARG operated. Can you describe what happened when it was time for a CARG member to visit the clinic and collect ART?
   Probes:
   Did the CARG members meet before the clinic visit?
   If yes, how was it organized? When and where did you meet? Who kept the client cards?How did you decide which CARG member conducts the clinic visit?
   Use of WhatApp?

5. Can you describe what happened after a CARG member visited the clinic to collect ART?
   Probes:
   How were the antiretroviral therapy drugs distributed?
   How did the process for distributing ART change over time?

6. How do people deal with money within the CARG?
   Probes:
   Payment for ART (if applicable)?
   Payment for CARG leader transport?
   Other money given to CARG leader?
   Have there been any problems? How where they handeled?

7. Please tell me about why you decided to leave your CARG?
   Probes:
   How long were you a member of the CARG?
   What contributed to your decision to leave the CARG?

8. Have any other members chosen to leave your CARG? If yes, do you know the reason(s) why?
   Probes:
   Does your former CARG still operate or did all members leave?
   Do you think your decision impacted the decision of other members?

9. How did being in a CARG change your ability to regularly take ART?
   Probes:
   How did being in a CARG make it easier or harder to get drugs from the clinic?
   How did being in a CARG make it easier or harder to regularly take your ART drugs?
   Have there been times other CARG members have delayed you receiving your ART and you have gone without drugs?

10. What did you like about being in a CARG?

11. What could be changed to make CARGs better?

12. What, if anything, would make you consider joining another CARG?
    Probes:
    What changes to CARG groups would make you consider joining another CARG?
    What changes to your current life situation would make you consider joining another CARG?

13. Please share how you are getting your ART treatment now? How often do you visit the clinic to collect ART?
    Probes:
    Overall, are you satisfied with the current way you receive your treatment? Why or why not?
    How could access to treatment be improved for you?

14. Are you aware of any other options offered by your facility to reduce the number of clinic visits needed to collect your ART?
    Probes:
    After asking the open-ended question, ask the following questions. However, only ask about the differentiated ART options offered at the site you are visiting:
    - Do you know if your facility offers multimonth ART dispensing: 3 month prescriptions for ART?
    - Do you know if your facility offers Fast-track refills: facility-based individual refill from a pharmacy?
    - Do you know if your facility offers Club refills: healthcare worker-led refill groups which meet in a room at the facility every 3 months?

- Do you know if your facility offers outreach ART services: Community-based individual ART delivered through mobile outreach every 3 months?
- Do you know if your facility offers family member refill groups: Similar to a CARG, but all members are part of a single family?

15. Ideally, how would you like to get your ART medicine?
    Probes:
    If you could create a system for picking up your ART drugs, what would it be? How would it work?
    Frequency of visits to get ART? Frequency seen by a nurse?

Form 4: Patient Interview Guide: Non-CARG Member

Instructions:

- The interview should be held in a private, quiet location.
- The interview should be conducted by a team of two: one who will lead the interview, and one who will take notes during the interview.
- A device needs to be available to record the interview, with extra batteries available in case they are needed.
- Before starting the interview, the client needs to have signed the Informed Consent Form:

  Verification of informed consent: ☐ YES ☐ NO

Introduction: Hello, my name is ____. Thank you for agreeing to talk to me today. I am here to talk with you as part of the I-TECH organization that supports the implementation of the Community ART Refill Group (CARGs) program in this district. As someone who is not a member of a CARG, your experience and thoughts are critical to improve the CARG program. We would like to thank you for agreeing to participate in this interview. Today, I would like to talk with you briefly about your knowledge and opinions of CARG membership and your suggestions for improving the CARG program.

1. To begin, I'm going to ask you a few questions about yourself and your experiences receiving care at this clinic.
   [*Administer Patient Participant Demographic Information Survey*]

2. Please tell me how you heard about CARGs?
   Probes:
   When did you first hear about CARGs?
   Have any healthcare workers discussed CARGs with you?

3. What did you hear were some of the benefits of joining a CARG?
   Probes:
   What aspects of joining a CARG appealed to you?
   Did the communal aspect of CARGs appeal to you? Why or why not?

4. What are the reasons why you have not joined a CARG?
   Probes:
   How did you make your decision?
   What were some of the barriers to joining a CARG?
   What were some of your concerns?

5. Is there any additional information you would like to know about CARGs?
   Probes:
   Would you have any questions for a healthcare worker?
   Would you have any questions for a current CARG member?

6. Do you know anyone enrolled in a CARG? What have you heard from their experiences?
   Probes:
   If you don't know anyone, what have you overheard in the clinic about CARGs?
   How did the experiences of others impact your decision about membership?

7. Can you see yourself joining a CARG in the future, why or why not?
   Probes:
   What changes to CARGs would make you more likely to join a CARG?
   Would you consider joining if you were the CARG leader/focal person?

8. Please share how you are getting your ART treatment now? How often do you visit the clinic to collect ART?
   Probes:

Overall, are you satisfied with the current way you receive your treatment? Why or why not?
How could access to treatment be improved for you?

9. Are you aware of any other options offered by your facility to reduce the number of clinic visits needed to collect your ART?

   Probes:

   After asking the open-ended question, ask the following questions. However, only ask about the differentiated ART options offered at the site you are visiting:

   - Do you know if your facility offers multimonth ART dispensing: 3 month prescriptions for ART?
   - Do you know if your facility offers Fast-track refills: facility-based individual refill from a pharmacy?
   - Do you know if your facility offers Club refills: healthcare worker-led refill groups which meet in a room at the facility every 3 months?
   - Do you know if your facility offers outreach ART services: Community-based individual ART delivered through mobile outreach every 3 months?
   - Do you know if your facility offers family member refill groups: Similar to a CARG, but all members are part of a single family?

10. Ideally, how would like to get your ART medicine?

    Probes:

    If you could create a system for picking up your ART drugs, what would it be? How would it work?
    Frequency of visits to get ART? Frequency seen by a nurse?

Form 5: Brief Satisfaction Survey for Focus Group Participants

Today's Date: ________________________ **Facility Name: _____________________________**

What is your age?

☐ 18-24 ☐ 25-34 ☐ 35-44 ☐ 45-54 ☐ 55 and above

What is your sex?

☐ Male ☐ Female

When did you begin working with HIV clients?
Month:___________ Year:______________

When did you begin working with CARGs?
Month:___________ Year:__________________

What is your position at the facility?

☐ Nurse ☐ Doctor ☐ Clerk ☐ Pharmacist
☐ Primary Counsellor ☐ CLF ☐ Other: ____________________

Are you seconded to this facility by I-TECH/UZCHS-CTU?

☐ Yes ☐ No

How do you believe CARGs have impacted the care of clients on ART?

☐ Large improvement in HIV care and patient outcomes
☐ Small improvement in HIV care and patient outcomes
☐ No change in HIV care and patient outcomes
☐ Small decline in HIV care and patient outcomes
☐ Large decline in HIV care and patient outcomes
☐ No opinion

Other Comments: ____

How have CARGs impacted your workload?

☐ CARGs have significantly reduced my overall workload
☐ CARGs have slightly reduced my overall workload
☐ CARGs do not impact my overall workload
☐ CARGs have slightly increased my overall workload
☐ CARGs have significantly increased my overall workload
☐ No opinion

Other Comments: ____

Do you agree that CARGs should continue to be supported at your facility?

☐ Strongly agree ☐ Agree ☐ Neutral ☐ Disagree ☐ Strongly disagree

Please explain why or why not: ____

Do you feel that you have received the training needed to support CARGs at your facility?

☐ Yes, I have received sufficient training ☐ No, I need additional training ☐ No opinion

If no, what additional training is needed? ____

Do you feel that you have received the resources needed to support CARGs at your facility?

☐ Yes, I have all the resources I need ☐ No, I need additional resource ☐ No opinion

If no, what additional resources are needed? ____

Do you anything else about how CARGs are managed in your facility that you wish to share? Any suggestions or ways to improve CARGs? ____

____

Form 6: Healthcare Worker Focus Group Discussions

Instructions:

- The focus group should be held in a private, quiet location.
- The focus group should be conducted by a team of two: one who will lead the focus group, and one who will take notes during the focus group.
- A device needs to be available to record the focus group, with extra batteries available in case they are needed.
- Before starting the focus group, all participants needs to have signed the Informed Consent Form:

  Verification of informed consent: ☐ YES ☐ NO

Introduction: Hello, my name is ____. Thank you for agreeing to talk to me today. I am here to talk with you as part of the I-TECH organization that supports the implementation of the Community ART Refill Group (CARGs) program in this district. We asked to speak with you today because we want to learn about your experiences working with CARGs, and your thoughts and experiences are critical to the implementation of the CARGs program. We would like to thank you for agreeing to participate in this focus group. We would like to learn about how CARGs are formed, how CARGs have affected your work, and how you see CARGs impacting the care of patients on ART. We encourage you to feel free to say anything concerning the topic of discussion. If a question is unclear to you, you can ask me to explain it. Your participation is voluntary and confidential.

1. To begin, when did each of you begin working at the facility [warm up question to get each person talking briefly]? Approximately when did the facility start having CARGs?

2. Can you describe how CARGs are typically formed?

Probes:
What role have health care workers played in encouraging and supporting clients to join CARGs?
Have you conducted any activities to encourage or assist clients to join CARGs?
How did patients respond to these activities? What was their initial interest in joining CARGs?

3. Are any types of patients more likely to be interested in joining a CARG? If so, why? Are any types of patients less likely to be interested in joining a CARG? If so, why?

Probes:
Men or women?
Older or younger?
Urban or rural?
Employed/unemployed?
Other categories of patients raised in previous interviews?

4. How have CARGs changed your job?

Probes:
In what ways has your job become easier?
In what ways has your job become harder?
Has it increased or decreased your workload?
[Try to differentiate between complexity (harder/simpler) and overall workload (ex. the total work hours required to provide care to 8 CARG members vs. 8 ART clients not in a CARG).]

5. How has patient care been impacted by CARGs?

Probes:
In what ways has the quality of patient care improved?
In what ways has the quality of patient care become worse or more difficult for clients?
Are you aware of any incidents where joining a CARG had a negative impact on a patient? (Example, a client not receiving drugs because of error by CARG leader)

Overall, do you feel that joining CARGs is beneficial, harmful, or neutral for patients on ART?

6. What are some of the barriers or challenges you face when implementing CARGs?
<u>Probes:</u>
Are there any problems or confusion around the current CARG forms [CARG Register, Monitoring Form, Supervision Visit Form]?
Are there any problems using the MOHCC registers when working with CARG members?
Have you been able to obtain annual viral loads from CARG members? And return viral load results to patients?
Would any additional support be beneficial?
Has care for CARG members changed over time? If so, in what ways?

7. How have CARG members been handling the user fees for receiving their ART (only if applicable)?
<u>Probes:</u>
Have problems arisen? If so, how have they been handled?

8. What is your opinion of the current eligibility criteria for joining a CARG?
<u>Probes:</u>
Are they too strict, so people who would benefit from CARGs are unable to join?
Are they too loose, so people who should not be joining a CARG are eligible?
Same geographical location? On ART for 6 months?

9. How are you communicating with CARG members given how infrequently they visit the clinic?
<u>Probes:</u>
Communication through CARG leaders?
Community outreach/tracking/follow-up?
Whatsapp?
Other methods?

10. Is there anything else that you would like to say about CARGs?
<u>Probes:</u>
Anything that we were discussing that you would like to add?
Or things you feel are important to share that we have no covered?

11. What other models for ART service delivery are offered by the clinic for stable clients?
<u>Probes:</u>
Multimonth ART dispensing: 3 month prescriptions for ART?
Fast-track refills: facility-based individual refill from a pharmacy?
Club refills: healthcare worker-led refill groups which meet in a room at the facility every 3 months?
Outreach: Community-based individual ART delivered through mobile outreach every 3 months?
Family member refill groups: Similar to a CARG, but all members are part of a single family?

12. As a final question, what do you think will happen with CARGs in the future?
<u>Probes:</u>
What will happen in the short term? Long term?
Will CARGs be sustained?
Will anything about CARGs need to be changed?
What would you like to see happen?

END OF SESSION: *"Now we have come to the end of our discussion. Thank you all so much for your participation!"*
